# Race-specific geography of prostate cancer incidence

**DOI:** 10.1186/1476-072X-5-59

**Published:** 2006-12-18

**Authors:** Laurie M DeChello, David I Gregorio, Holly Samociuk

**Affiliations:** 1 Department of Community Medicine & Health Care, University of Connecticut School of Medicine, 263 Farmington Ave, Farmington, CT 06030-6325, UK

## Abstract

**Background:**

This study evaluated geographic distribution of race-specific prostate cancer incidence in Connecticut and Massachusetts. This cross-sectional analysis of census and cancer registry data included records of 29,040 Whites and 1,647 African Americans diagnosed with incident prostate cancer between 1994 and 1998. A spatial scan statistic was used to detect and test significance of the geographic variation in race-specific incidence rates within the two-state area.

**Results:**

Significant geographic variation in age-adjusted incidence rates among both White and African American men was observed, with little overlap noted between distributions. Identified locations reflected patterns of residential segregation and socio-economic conditions. Among Whites, places with higher than expected incidence had higher socioeconomic status than places with lower than expected incidence. No discernable relationship between social indicators and rate variation among African Americans was evident.

**Conclusion:**

Differences in race-specific geographic distribution of prostate cancer incidence do not suggest a shared environmental etiology. Furtherstudyof genetic, behavioral and health carefactors affecting the occurrence and/or reporting of the disease is warranted. This study highlights the need for race- and geographic-specific interventions to better control disease within at-risk communities and for on-going analysis into social and contextual factors that contribute to observed disparities between African Americans and Whites in the occurrence of cancer.

## Background

Despite, or perhaps because of, the high frequency of prostate cancer incidence in the United States (it is the most common non-dermatological malignancy diagnosed among American men), we remain unsure about the cause(s) and ways to effectively control the disease in the population [1]. Genetic susceptibility, as well as age, dietary practices, physical activity, agrochemical exposures, infectious diseases, and socioeconomic status (SES) have been suggested as risk factors for disease[2] and/or disease detection [3-6].

The profile of prostate cancer among African American men, in particular, is sufficiently dissimilar to that of Whites as to suggest, on the one hand, a distinct etiology and disease course[7], and on the other hand, disparities between groups in health practices and medical care delivery/utilization. Prostate cancer incidence is 60% greater among African Americans than similarly aged Whites in the United States [8]. Diagnosis with advanced disease is more prevalent among African Americans, contributing to a prostate cancer mortality rate that is twice the rate among Whites [9]. Their age-adjusted prostate cancer mortality rate places African Americans among the most severely affected worldwide [8].

Geographic studies of cancer incidence can provide important guidance for disease control and prevention practices by highlighting high risk communities in need of enhanced interventions [10]. Location (e.g., one's place of work or residence, proximity to purported hazards, etc.) accounts for dissimilarities in the composition of populations, differentiates risks/protections that are the product of physical and social environments, and affects the transfer of ideas, resources and behaviors among and between groups through social-cultural variability [11]. As such, geographic analysis of prostate cancer incidence rates may be a source of hypotheses about carcinogenesis and/or preventive and clinical service delivery and utilization [12].

Several geographic studies of prostate cancer can be cited [5,13-16], but relatively few examine incidence patterns with specific attention to the race of cases [17-19]. In Virginia, the geographic distributions of White and African American cases appeared similar[17], while in Louisiana, marked differences were observable [18]. Within Maryland, the racial composition of census block groups was found to account for variation in the proportions of high grade and late stage tumors detected there [19].

This paper contributes to the ongoing description of race-specific variation in the geography of prostate cancer incidence and addresses whether differences exist between observed incidence patterns between Whites and African Americans with whom they share environments in common. The geographic variation of race-specific prostate cancer incidence diagnosed between 1994 and 1998 in Connecticut (CT) and Massachusetts (MA) residents was evaluated.

## Results

**Figure 1:**
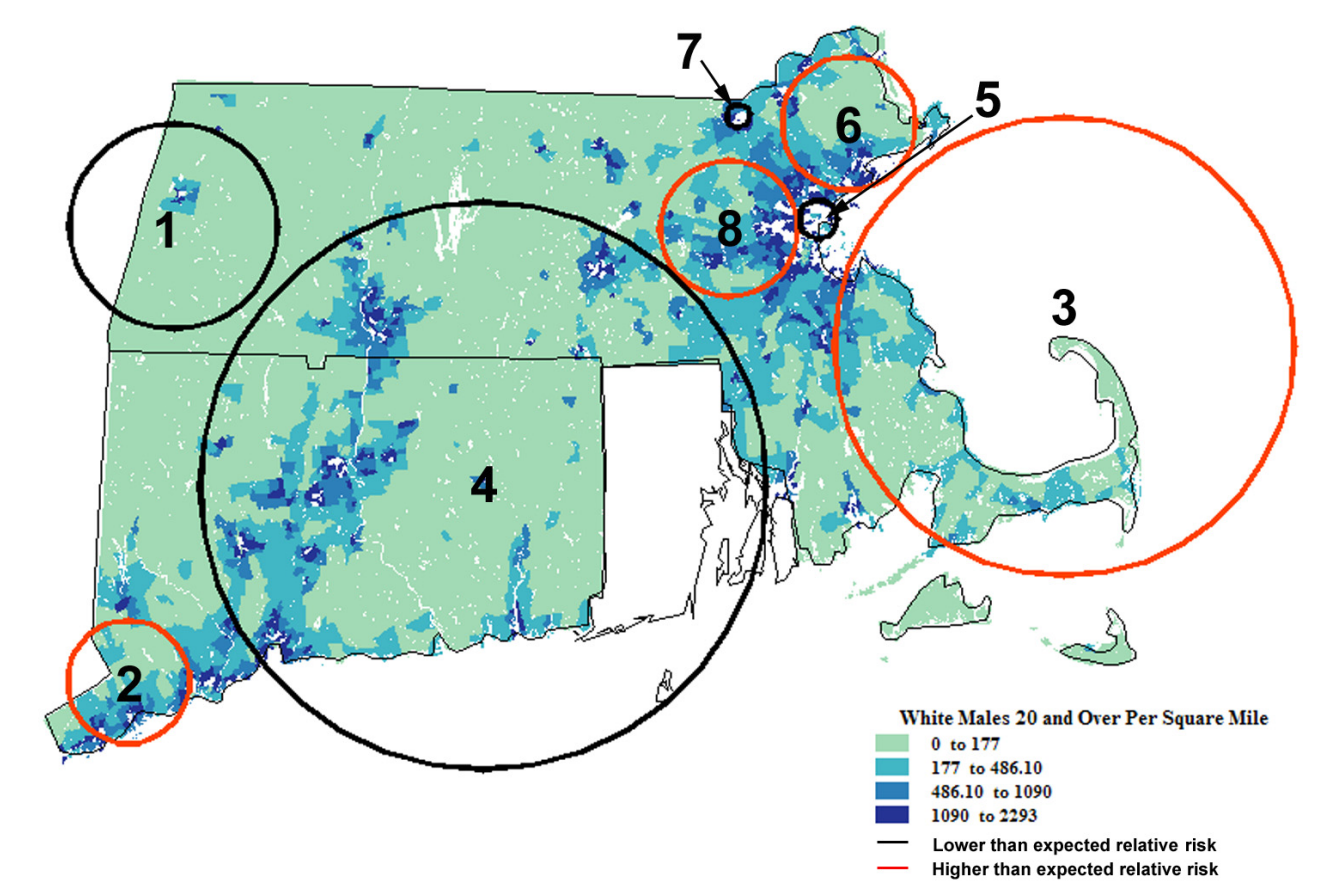
**Spatial analysis of White residents of Connecticut (CT) and Massachusetts (MA)**. Population density† (1990) and geographic variation of age-adjusted invasive prostate cancer incidence rates (1994–98) among Whites residents of CT and MA. †Men 20+ years old per square mile.

**Figure 2:**
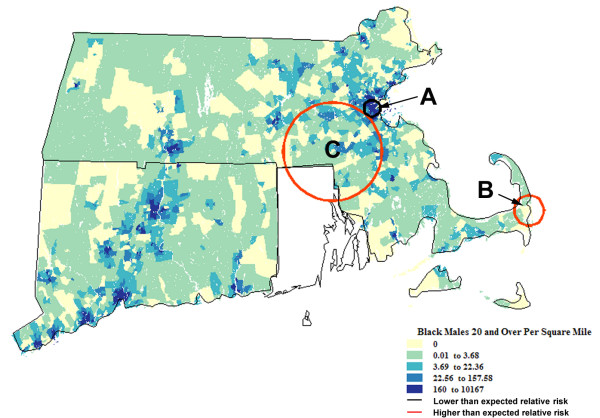
**Spatial analysis of Black residents of Connecticut (CT) and Massachusetts (MA)**. Population density† (1990) and geographic variation of age-adjusted invasive prostate cancer incidence rates (1994–98) among Black residents of CT and MA. †Men 20+ years old per square mile.

Population densities specific to Whites and African Americans across CT and MA are presented in Figures 1 and 2, respectively. Populations of both groups were concentrated in and around Boston, along the Connecticut River Valley of Central Connecticut and Western Massachusetts, and along the Connecticut shoreline. Whites were evident in urban, suburban and rural communities throughout the study area whereas African Americans were predominantly urban dwellers and absent from many suburban and rural locations. Also, African American were roughly 3-times as likely as Whites to have lived in poverty, twice as likely to have been unemployed or undereducated (i.e., < 12 years schooling) and half as likely to have owned the dwelling where they lived.

Across the two-state study area, the average annual age-adjusted rate of invasive prostate cancer among Whites during 1994–1998 was 197.0 cases/100,000/year (1990 standard population). The spatial scan statistic identified 8 locations (illustrated by bold circles in Figure 1 with summary statistics in Table 1 and numbered in order of statistical significance) where incidence rates were estimated to differ significantly from the null as reflected by the overall two-state age-adjusted incidence rate. The most probable location of 'true' rate variation was in Western MA (Zone 1) where the observed average annual age-adjusted disease rate of 70.0 cases/100,000/year was 0.355-times what was expected compared to experiences elsewhere in the study area (*P *< 0.0001).

**Table 1:** Statistics of the spatial analyses.

Zone	Population At Risk	Observed Cases	Observed/Expected	p-value
Analysis specific to White males				
1	36,185	146	0.36	<0.0001
2	107,046	1,517	1.34	<0.0001
3	190,612	2,509	1.24	<0.0001
4	1,086,721	10,020	0.92	<0.0001
5	119,552	818	0.73	<0.0001
6	155,913	1,898	1.24	<0.0001
7	26,704	120	0.51	<0.0001
8	275,481	2,916	1.14	<0.0001
Analysis specific to African American males				
A	32,522	267	0.72	<0.0001
B	21	6	19.24	0.0168
C	6,961	94	1.70	0.0244
Analysis specific to African American males after adjustment for spatial distribution of rates specific to White males				
a	31,416	260	0.75	0.0022
b	1,731	43	2.35	0.0134

Statistics of the primary and statistically significant secondary clusters from the spatial analyses of white and black male prostate cancer incidence in CT and MA, 1994–1998.

By comparison, the observed incidence rate among White men living in southwestern CT (Zone 2) was 1.337-times greater than that of men living elsewhere within the study area (*P *< 0.0001). Tracts on and adjacent to Cape Code (Zone 3), and communities west and northeast of Boston (Zones 8 and 6, respectively) were found to have incidence rates that differed significantly (high), while a substantial portion of central and eastern CT (Zone 4) and 2 circumscribed locations of northeastern MA (Zones 5 and 7) exhibited rates that differed markedly (low) from expectation. Taken together, the populations within Areas 2, 3, 6 and 8 encompassed 729,052 persons (24.7% of all at-risk men) and 8,840 invasive cases (30.4%); those locations with attenuated disease rates (Zones 1,4,5 and 7) affected 1,269,162 persons (43% of all at-risk men) and 11,104 (38.2%) reported cases.

Across CT and MA, the average annual age-adjusted rate of invasive prostate cancer among at-risk African Americans for 1994–98 was 203.0 cases/100,000/year (1990 standard population). The pattern of geographic variation specific to African Americans differed in several important respects from that of Whites. Among the 3 identified locations, which are lettered in order of statistical significance (See Figure 2), two zones were estimated to have had rates significantly higher than expectation based on the overall experience of African Americans within the two-state study area. The most likely zone of true variation occurred in the Boston area (Zone A) where African Americans had 28% lower risk than those living elsewhere (P < 0.0001). Zone B on Cape Cod and Zone C in eastern MA exhibited excess incidence relative to other locations within the study area.

The consequence of these patterns, however, was considerably less among African Americans than Whites. There were no locations of significant variation in CT among African Americans. The two locations with elevated disease rates pertained to only 5.7% of diagnosed cases among African Americans and 4.0% of the at-risk population; the area with lower than expected incidence pertained to 15.2% of all cases and 18.8% of the total population-at-risk.

Lastly, we re-examined incidence among African Americans with further adjustment of rates acknowledging geographic variation of incidence specific to Whites (i.e., Zones 1–8 above). Doing so yielded information about places where risks of prostate cancer both differed significantly across African American communities, as well as African Americans and Whites living within similar locales. The zones are lettered in order of statistical significance and use lower case to differentiate them from the preceding analysis.

**Figure 3:**
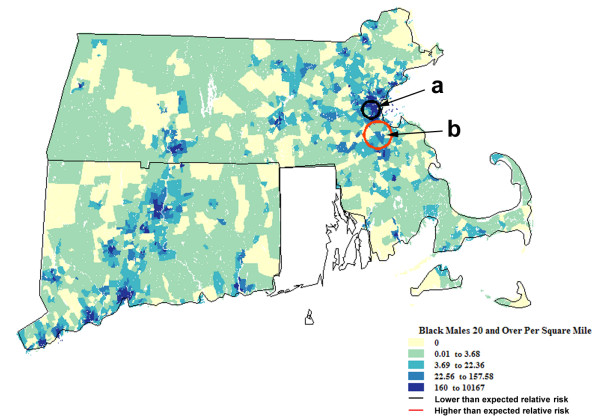
**Spatial analysis of Black residents of Connecticut and Massachusetts adjusted for White resident analysis**. Population density† (1990) and geographic variation of age-adjusted invasive prostate cancer incidence rates (1994–98) among Black residents of CT and MA, adjustment for spatial distribution of incidence rates specific to Whites. †Men 20+ years old per square mile.

The spatial scan statistic identified two places (see Figure 3) where average annual incidence rates among African Americans, adjusted for patient age and the underlying white incidence rates, differed significantly from the null. These findings suggest locations where incidence of prostate cancer differs both from African Americans living elsewhere in the study area, as well as White men living within the identified locales. Zone a was roughly equivalent to Zone A in the preceding analysis. The persistence of this location suggests that incidence among African Americans largely was independent of factors that contributed to rate variation among White neighbors. Places of markedly high or low incidence rates among White men were not measurably associated with observed rate variation among African Americans. Zone B from the analysis of African Americans alone is not significant when African Americans are adjusted for the rates of their White neighbors. Zone b in this analysis is a subset of Zone C from the previous analysis.

Some investigating was done to ensure that there were not smaller zones within the analysis of Whites similar to zones found in the analysis of African Americans that were covered up by more likely larger zones. Tracts that were in significant zones from the African American analysis were identified; the age adjusted relative risks for White men in these tracts were calculated. All of these zones had relative risks lower than 1 in White males.

## Discussion

Although the national rate of prostate cancer incidence in African American men is much higher than in White men, we reported race-specific rates for our study area that were not very different. Race- and state-specific rates were calculated; African American men in CT had about 18 cases/100,000/year higher rate than CT White men, but MA African American men had about 7 cases/100,000/year lower rate than MA White men. This accounts for why there is not much difference between the race-specific rates of the 2-state study area.

Striking variation in geographic distribution of prostate cancer incidence was noted for both Whites and African Americans. Moreover, differences between analyses suggested little commonality of experiences between groups. Not only do the figures of White males and African American males look dramatically different, but the investigation of White males in the significant African American zones all had RRs of about one. While Whites and African Americans share much of the physical, economic and social environments of these two New England states, these findings suggest that important differences between groups pertaining to residential patterns and socio-economic forces modify the risks of disease (and its detection) that neighbors, even those living in close proximity, face as a consequence of their racial identities. Findings of relatively high/low rates of disease specific to one group did not extend to the other at that same location. Such results cast doubt on the likelihood that a common environmental etiology was at work affecting men regardless of racial background, in favor of explanations that favor genetic and/or behavioral causes specific to one or another group. Economic and health system factors may differentially influence detection of cases between population groups. In particular, longstanding patterns of residential and economic segregation may account for findings reported here.

Among Whites, places identified in the preceding analysis as having had disease rates that exceeded expectation were segregated places which differed from other locales within the study area. For example, such places exhibited relatively high levels of education, economic status, home ownership and employment, relative to identified places with incidence rates below expectation (refer to Table 2). On the other hand, an effect of relative deprivation on disease rates among Blacks, for whom the range of residential and socio-economic disparities may be less pronounced, was not readily discernable since there were only two locations identified as significantly different compared to the rest of the study area. However, the tracts in the zone of higher than expected risk in African Americans did have higher socioeconomic indicators than the zone with lower than expected risk. These analyses suggest that the potential effect of geographically-based differences of lifestyles may point to variation in their relative likelihood of developing prostate cancer. At least across White communities, it appears that relative affluence is associated with elevated disease incidence.

**Table 2:** Socioeconomic statistics of identified clusters.

Zone	% Black Residents*	% less than 12 years schooling^†^	% below poverty^†^	% renter occupied dwellings^‡^	% unemployed^§^
Whites with incidence greater than expectation					
2	4.5	12.3	3.1	22.3	3.9
3	1.7	12.6	4.8	24.9	6.6
6	1.4	14.5	5.0	31.5	5.6
8	2.8	10.9	5.3	40.1	4.8
Whites with incidence less than expectation					
1	1.6	19.8	6.8	31.6	6.2
4	5.8	21.6	5.9	35.4	5.5
5	4.4	24.5	10.1	63.5	7.0
7	2.2	33.4	10.2	54.2	10.2
African Americans adjusted for Whites with incidence greater than expectation					
b	3.0	14.6/11.6	4.1/3.9	29.1/44.6	6.1/6.1
African Americans adjusted for Whites with incidence less than expectation					
a	19.1	18.0/34.5	15.5/25.4	70.4/82.8	7.1/13.9

Socioeconomic characteristics of identified locales with significant variation of prostate cancer incidence by race, CT and MA, 1994–1998. 1990 Census data for is of White and African American residents; the second percentage in the African American zones are of African American residents alone.

*Denominator includes male White and African American residents from the 1990 census 20 years of age and older. ^†^Persons 18 years and older. ^‡^Occupied housing units. ^§^Persons 16 years and older.

## Conclusion

Despite considerable evidence of geographic variation in prostate cancer incidence [5] and mortality [20], few pertinent clues regarding etiology have emerged. A source of difficulty is the challenge of differentiating factors relevant to disease incidence vis-à-vis its detection. This population-based study of race-specific variation of prostate cancer incidence across Connecticut and Massachusetts illustrates several important differences in the spatial distribution of cases within and between these groups. Such dissimilarities reason against environmental factors as a root cause of disease and favor genetic, behavioral and health care explanations for local variations. In particular, residential segregation and socio-economic inequities may exert powerful influences on the likelihoods that men develop and are diagnosed with the disease. As such, this study calls for race and geographic-specific understanding to better control disease within at-risk communities.

## Methods

Geographic variation in race-specific incidence of prostate cancer (ICD-O-2: C61.9) across Connecticut (CT) and Massachusetts (MA) was assessed by comparing the distributions of geocoded cases (i.e. records for which a census tract of residence at the time of diagnosis was discernable) to the counts of at-risk males residing at those locations. Census tracts provide a sensitive analysis of densely populated areas and are more homogeneous in their resident characteristics than towns or Zip Code areas [21]. The University of Connecticut, CT State Department of Public Health, and MA Department of Public Health institutional review boards approved the access and analysis of CT Tumor Registry (CTR) and MA Cancer Registry (MCR) data.

The combined 1990 Census Summary Tape File 1 for CT and MA identified 2,949,032 White and 203,340 African American men 20 years of age and older within 2,165 census tracts [22]. For each geographic location, race-specific population counts were organized by race according to 7 age categories (i.e., 20 to 29, 30 to 39, 40 to 49, 50 to 59, 60 to 69, 70 to 79, and 80+ years).

Between 1994 and 1998, invasive prostate cancers were diagnosed in 31,767 White and 1,916 African American residents of the 2-state study area. Of these, we identified the census tract of residence of 29,040 White (91.4%) and 1,759 African American (91.8%) cases. It was necessary to exclude records of 2,727 Whites and 157 African Americans from analysis because they lacked sufficiently accurate address information to assign a census tract location or they specified an indeterminate location (i.e., P.O. Box). An additional 112 African American records were excluded (38 from CT and 74 from MA) because a case's purported census tract of residence lacked comparable persons at-risk residing within that location. Our analyses pertain to records of 29,040 White and 1,647 African American cases.

### Statistics

Geographic variation of incident cases was evaluated using a spatial scan statistic [23]. The procedure employed a large number of scanning circles that varied by size (encompassing up to 50% of the study area's at-risk population) and location (any and all map coordinates within the study area) to compare the distribution of observed cases to the number expected, according to a null hypothesis that incidence was proportional to the population densities of specific locations. The likelihood that measured variation was detected by chance was evaluated in relation to observed findings of 9,999 Monte Carlo simulations with adjustment of *P*-values for multiple testing inherent in the surveillance procedure. The Poisson probability model was utilized in this method.

The spatial scan statistic is well suited to geographic surveillance as it accounts for uneven geographic distributions (both high and low) of cases and population densities. It does so without a priori assumptions as to the size or location of potential variation and can adjust findings for relevant (e.g., age) covariates. Results of the spatial scan statistic are considered to be conservative estimates of the likelihood of observing events within a specified zone, relative to places elsewhere around the study area [24]. The spatial scan statistic was calculated using *SaTScan 6.1 *[25] and results are illustrated using *Maptitude 4.5 *software [26].

First, age-adjusted *SaTScan *analyses specific to Whites or African Americans are presented. We subsequently evaluated geographic variation of age-adjusted incidence among African Americans with further rate adjustment to account for geographic variation of observed incidence among Whites. Linking data files in this way allows simultaneous consideration of how incidence rates vary across the African American population, as well as between the Whites and African Americans. Lastly, to begin to place findings into proper context, we characterize places with marked variation of incidence rates in relation to key socio-economic indicators that may have underscored the likelihood of disease and/or its detection at those locations (i.e., percentages of persons living below poverty, having less than 12 years of schooling, living in non-owner-occupied dwellings and unemployed) [5,27].

## Authors' contributions

LMD participated in design of the study, performed the statistical analyses and drafted the manuscript. DIG conceived of the study, participated in design of the study, and aided in drafting the manuscript. HS aided in data preparation, analyses and drafting of the manuscript. All authors read and approved the final manuscript.
